# Buffalo Medical and Surgical Association

**Published:** 1882-09

**Authors:** C. M. Daniels

**Affiliations:** Secretary


### Buffalo Medical and Surgical Association.

*Regular Meeting, August 7th, 1882.*

President, Dr. A. R. Davidson, in the chair. Dr. C. M. Daniels, Secretary.

Present—Drs. Cronyn, Wm. Ring, Mynter, Samo, Burghardt, Rochester, Runner, Mann, Hauenstein, Gay, C. A. Ring, Hopkins, Kamerling, Bartow, Dorland, Fowler, Cary, Hartwig, Strong, Moody, Brecht, Macniel, Bartlett and Barnes.

By invitation, Drs. Heath, Frank, Long, Berkes, Potter, Frederick, Tremaine, and DeLancey Rochester.

Election of new Members—Drs. M. D. Mann and Chas. A. McBeth.

*Essay*—By Dr. M. D. Mann, subject: "Management of Abortion." The paper appears complete in the September number of the Buffalo Medical Journal.

*Discussion*—Dr. Cronyn thought the directions given hardly left room for discussion. He firmly believed in the early removal of the placenta, also favored ergot and quinine; recommended the tampon and related personal experiences.

Dr. Ring said accidental miscarriages were troublesome affairs; referred to the kite-tail tampon and endorsed the use of the water bag when available. He related a case where the only styptic at hand was alum, which was carried into the vagina and proved efficacious. He had since tried the pulverized alum in the vagina, followed by tampon, about a dozen times with the greatest satisfaction. He also related a case where placenta was retained for ten days when it was forcibly removed and found in excellent condition.

Dr. Hauenstein had been much interested and thought the paper fully covered the ground. He alluded to the different ideas among physicians as to the best means of controlling hemorrhage. He described tampon as used by himself, also the rubber ball, or colpeurynter, which may be dilated by air or water, which has been most satisfactory in his hands.

Dr. Rochester agreed heartily in all the recommendations. He said that Dr. Gilman taught that opium would sometimes produce abortion and the speaker's experience had led him to believe that this was sometimes the case. He thought the fluid ext. Hamamelis very valuable to prevent hemorrhage, also endorsed quinine as an ecbolic. Related case of a woman who had been delivered at seventh month, but placenta retained a full month; removal by the hand was impossible until dilatation by large sponge tent. The woman became pregnant again within a year.

Dr. Strong spoke in favor of the kite-tail tampon and supported Dr. Rochester as to the antiseptic property of quinine and its value in preventing suppuration.

Dr. Bartlett related a case where he had employed a lamp chimney as a substitute for the speculum in introducing tampon, the case being urgent and the proper instrument unobtainable.

Dr. Mann spoke against the antiseptic action of quinine and mentioned its action on certain fluids, white blood corpuscles, etc. The result, however, was dependent upon the presence of a considerable quantity and that we could not get enough into the blood without killing the patient.

Dr. Cronyn said he did not mean the antiseptic action of quinine, but that together ergot and quinine act better than either alone.

*Prevailing Diseases*—Dr. Rochester reported that the last week had brought cholera infantum. He had had some severe cases terminating fatally in from two to five days, with peculiar head symptoms.

Dr. Bartlett mentioned diarrhœa and dysentery as being almost epidemic.

Dr. Cronyn had noticed the peculiar odor of sulphuretted hydrogen in the dejections.

Dr. Hauenstein had seen some scarlet fever, a good deal of cholera infantum and one severe case of cholera in an adult, presenting the characteristic symptoms of Asiatic cholera.

Dr. Ring had seen many cases of cholera infantum. He referred briefly to the usual treatment and said that where the mother's milk was deficient he thought well of condensed milk; also spoke highly of the milk supplied from Jewett's dairy farm.

Dr. Moody also endorsed Jewett's milk.

*Voluntary Communications*—Dr. Hartwig presented a case of strumous disease of the knee-joint, from which he had removed foreign bodies after free incision, as explained in a previous meeting, and asked what further could be done in the case.

Dr. Gay said, that his namesake, of London, first proposed the operation; that he had practiced it many times; had operated at the Alms House for the same trouble as in case described by Dr. Hartwig.

Dr. Mynter thought foreign growths should be removed from suppurating joints, but that joints should not be opened for the purpose of diagnosis.

Dr. Tremaine thought knees should not be opened, except in those cases which had been mentioned by Dr. Mynter. He had seen a perforating gun-shot wound of patella from 45 calibre gun, penetrating to head of tibia, followed by complete recovery. But one or several cases of this kind did not give a warrant for operative interference with the joint.

Dr. Hartwig remarked that he did not open the joint for the purpose of diagnosis, but first used a trocar, then followed with the operation for the removal of the bodies in the joint.

*Reports of Committee*—The President called for a report from the committee appointed at a previous meeting "Upon charges preferred against certain members of the Association for non-payment of dues for more than five years." The report of the committee recommended that of physicians resident in the city the names of four should be dropped from the list, and that the names of seven others, not now residing in the city, but who have omitted to send in their resignations, should be erased from the list of members.

After some discussion the recommendation of the committee was adopted.

Adjourned. C. M. Daniels, *Secretary*.

